# Polygenic scores in Familial breast cancer cases with and without pathogenic variants and the risk of contralateral breast cancer

**DOI:** 10.1186/s13058-025-02107-5

**Published:** 2025-09-08

**Authors:** Anders Kvist, Anders Kämpe, Therese Törngren, Bianca Tesi, Panagiotis Baliakas, Åke Borg, Daniel Eriksson

**Affiliations:** 1 Division of Oncology, Department of Clinical Sciences Lund, Lund University, Lund, Sweden; 2 Department of Molecular Medicine and Surgery, Karolinska Institutet, Stockholm, Sweden; 3 Institute for Molecular Medicine Finland (FIMM), University of Helsinki, Helsinki, Finland; 4 Department of Medicine Huddinge, Center for Hematology and Regenerative Medicine (HERM), Karolinska Institutet, Stockholm, Sweden; 5 Clinical Genetics and Genomics, Karolinska University Hospital, Stockholm, Sweden; 6 Department of Immunology, Genetics and Pathology, Uppsala University, Uppsala, Sweden; 7 Clinical Genomics Uppsala, Science for Life Laboratory, Uppsala University, Uppsala, Sweden

## Abstract

**Background:**

Polygenic risk scores (PRS) are not yet standard in clinical risk assessments for familial breast cancer in Sweden. This study evaluated the distribution and impact of an established PRS (PRS_313_) in women undergoing clinical sequencing for hereditary breast cancer.

**Findings:**

We integrated PRS_313_ into a hereditary breast cancer gene panel used in clinical practice and calculated scores for 262 women. Comparisons were made between women with unilateral and contralateral breast cancer, as well as those with and without pathogenic variants in breast cancer susceptibility genes. PRS_313_ was significantly higher in women with contralateral breast cancer (median + 1.3 SD, *n* = 33, *P* = 8e-9) compared to those with unilateral disease (median + 0.66 SD, *n* = 197, *P* = 5e-10). Elevated PRS_313_ was also observed in women with pathogenic variants, including those in high-penetrance genes (+ 0.65 SD) and moderate-penetrance genes (+ 0.93 SD), compared to population controls. Incorporating PRS_313_ into a clinical risk model (BOADICEA), shifted 20%-27% of women with moderate-penetrance variants and 23%-32% of women without pathogenic variants into different risk categories according to NCCN and NICE guidelines.

**Conclusions:**

Women with familial breast cancer showed elevated PRS_313_, including those with pathogenic variants, contributing to the observed high risk in these families. Integrating PRS into risk assessment and genetic counselling has the potential to refine risk predictions, even among women with breast cancer attributed to monogenic variants.

**Supplementary Information:**

The online version contains supplementary material available at 10.1186/s13058-025-02107-5.

## Introduction

Breast cancer (BC) is one of the most common cancers worldwide [1, 2]. Familial BC, characterized by multiple cases in a family or early disease-onset, is suggestive of an underlying genetic predisposition. The identification of a significant genetic determinant could motivate personalized surveillance programs for healthy relatives, or even bilateral risk-reducing mastectomy, motivating genetic investigations. Evaluation of index patients includes testing for pathogenic variants in approximately 12 genes, such as *BRCA1* and *CHEK2* [3–6]. However, these rare monogenic cancer syndromes sought in clinical routine account for fewer than 10% of familial BC cases, leaving most families without an identifiable inherited cause [7].

Genome-wide association studies have identified several hundred common risk alleles associated with breast cancer. The effect of each of these genetic variants on BC risk is typically small and individually they are of negligible relevance in clinical practice. However, together the common variants explain a significant proportion of BC risk, beyond the contribution of rare high-penetrance variants [8, 9].

Polygenic risk scores (PRS) are tools that aggregate the effects of numerous common genetic variants, each making a small contribution to disease risk, into a single metric that estimates their overall effect on an individual’s inherited predisposition to a given condition. The predictive performance of PRS has been validated in large prospective cohorts, demonstrating their utility for stratifying individuals by risk level and improving personalized risk estimates [10–14]. For example, women in the highest percentile of PRS exhibit an absolute lifetime risk of BC exceeding 30%, a threshold that, according to the UK National Institute for Health and Care Excellence (NICE) guidelines, warrants consideration of bilateral risk-reducing mastectomy [4].

In families carrying rare pathogenic variants in established BC susceptibility genes, these common risk alleles can further modify disease penetrance and lifetime risk, which could impact clinical management in this group as well [5, 6, 15]. Nevertheless, PRS is not routinely used in the clinical evaluation or risk estimation for affected women and their family members. However, its potential to refine risk assessment, particularly when integrated with traditional risk factors and monogenic findings, makes it a promising tool for individualized care.

The BOADICEA (Breast and Ovarian Analysis of Disease Incidence and Carrier Estimation Algorithm) model is a validated tool for breast cancer risk prediction that integrates PRS along with other risk factors, and is available through the CanRisk tool [16, 17]. PRS contributes the widest distribution of predicted risk among all factors in the model, but the highest predictive accuracy is achieved when all risk factors are used jointly.

In this study, we assessed the PRS_313_ for risk prediction of 262 Swedish women with familial BC, undergoing genetic testing according to the National Clinical Cancer Care program [3]. We added the 313 common variants into the clinical sequencing panel and calculated individual-level PRS. We compared personalized risk management recommendations made *without* and *with* the PRS_313_, according to guidelines from the US and the UK (National Comprehensive Cancer Network^®^ (NCCN) and NICE, respectively). Our findings provide a real-life validation of the effects of PRS on clinical decision-making.

## Results

### Study participants and BC characteristics

We included 262 women undergoing evaluation for familial BC. All participants received genetic counselling, during which family history and tumor pathology information were collected. In addition to testing established BC genes, the PRS_313_ was included into the genetic screening. For comparison, we used PRS data from 1,000 Swedish population controls (SweGen), which exhibits population parameters comparable to CanRisk, the tool employed for risk estimation [17, 18]. All included participants were of broadly European descent (Suppl. Fig S1) [18].

**Fig. 1:**
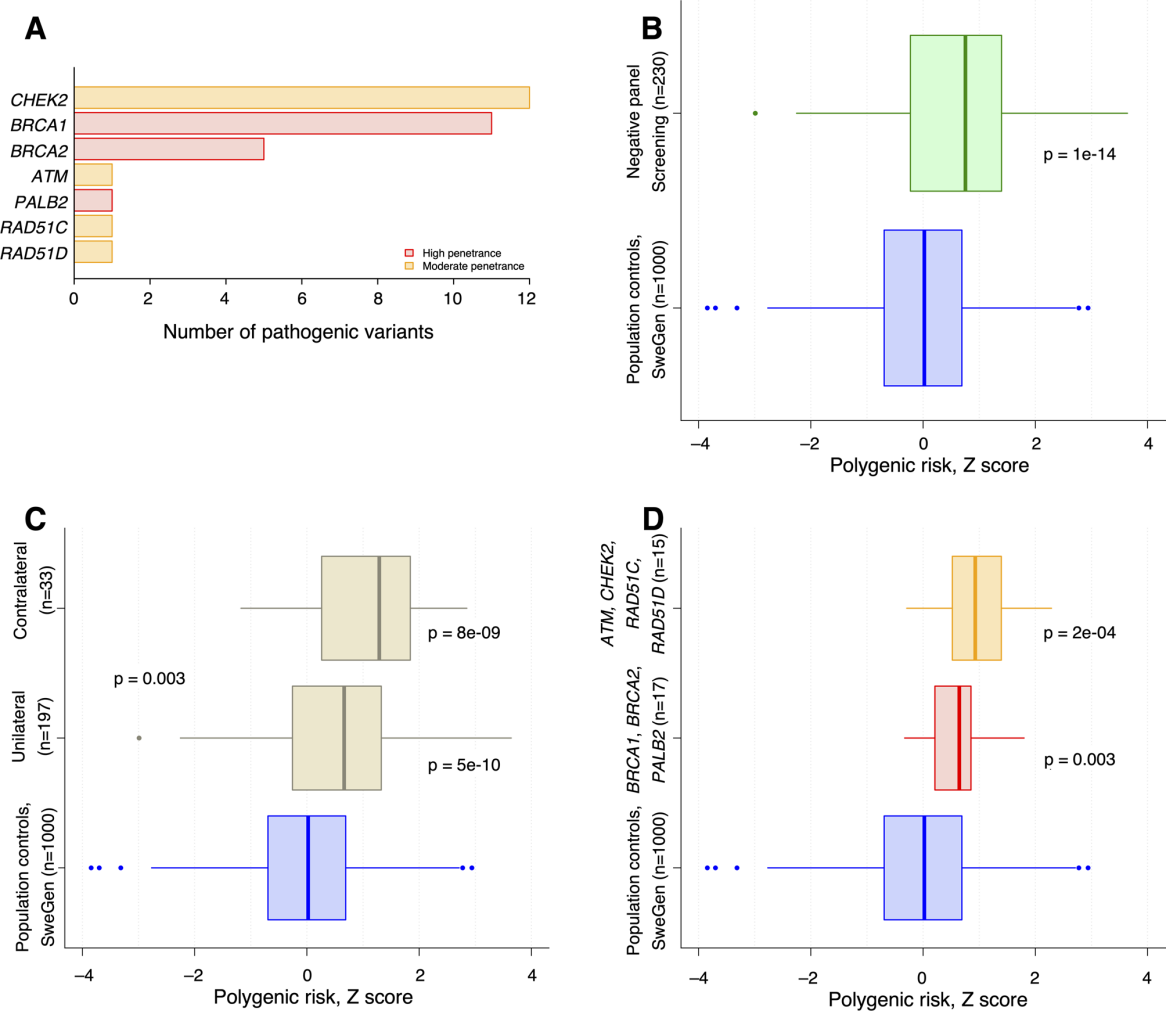
**Panel A**: displays the breast cancer genes in which at least one pathogenic variant was found, and bars represent the number of women with a pathogenic variant in each of the genes. **Panel B**: shows the PRS_313_ distribution in women referred for familial BC, in whom no pathogenic variant could be found (*n* = 230). In comparison with population controls (SweGen, *n* = 1000), there was a statistically significant overrepresentation of high PRS_313_ (*P* = 1e-14, Mann-Whitney). The scores have been normalized so that the population controls have mean 0 and standard deviation 1. **Panel C**: presents the distribution of PRS_313_ in women without a detected pathogenic variant (*n* = 230), further stratified by unilateral (*n* = 197) and contralateral (*n* = 33) BC. Statistically significant differences were observed between all groups. **Panels D**: displays the distribution of PRS_313_ in women with pathogenic variants in established breast cancer genes, with genes categorized into moderate-penetrance (*n* = 15) and high-penetrance (*n* = 17) groups. Both categories showed statistically significant differences compared to population controls

The mean age of first BC was 50 years (range 24–80) with 14% of the women having contralateral BC at the time of counselling (Suppl. Table S1, Fig. S2). The average age at contralateral BC was 61 years (range 28–77). Of all tumors, 13% were negative for hormone receptors and HER2. Among the 262 women, 230 had a negative panel, while 32 had a pathogenic variant in the following genes: *ATM* (*n* = 1), *BRCA1* (*n* = 11), *BRCA2* (*n* = 5), *CHEK2* (*n* = 12), *PALB2* (*n* = 1), *RAD51C* (*n* = 1), *RAD51D* (*n* = 1) (Fig. 1A, and Suppl. Table S2).

### Distribution of PRS_313_ in women with familial BC

Compared to population controls (median Z-score = 0), patients with familial BC but no pathogenic variant showed higher PRS_313_ (median + 0.75 SD, *P* = 1e-14, *n* = 230), corresponding to the 66:th percentile (95% CI [63%, 70%]) (Fig. 1B). Seven out of ten women had a PRS_313_ above the population average, and one out of nine had a PRS_313_ more than 2 SD above average. Stratifying women without pathogenic variant by side involvement, we observed higher PRS_313_ both among women with unilateral BC (median + 0.66, *P* = 5e-10, *n* = 197), as well as contralateral BC (median + 1.3, *P* = 8e-9, *n* = 33) (Fig. 1C). In addition, the PRS_313_ was higher in contralateral compared to unilateral cases (*P* = 0.003).

Looking only at the 32 women where a pathogenic variant was identified, we observed increased PRS_313_ both in women with a pathogenic variant in high-penetrance genes (*BRCA1*, *BRCA2*, *PALB2*) (median + 0.65 SD, *P* = 0.003, *n* = 17), as well as women with a pathogenic variant in a moderate-penetrance gene (*ATM*, *CHEK2*, *RAD51C*, *RAD51D*) (median + 0.92 SD, *P* = 2e-4, *n* = 15) (Fig. 1D). On average, women with a high-penetrance pathogenic variant belonged to the 71:st percentile of PRS_313_ (95% CI [62%, 81%]), women with a moderate-penetrance variant belonged to the 78:th percentile of PRS_313_ (95% CI [68%, 88%]).

### Lifetime risk of BC

**Fig. 2:**
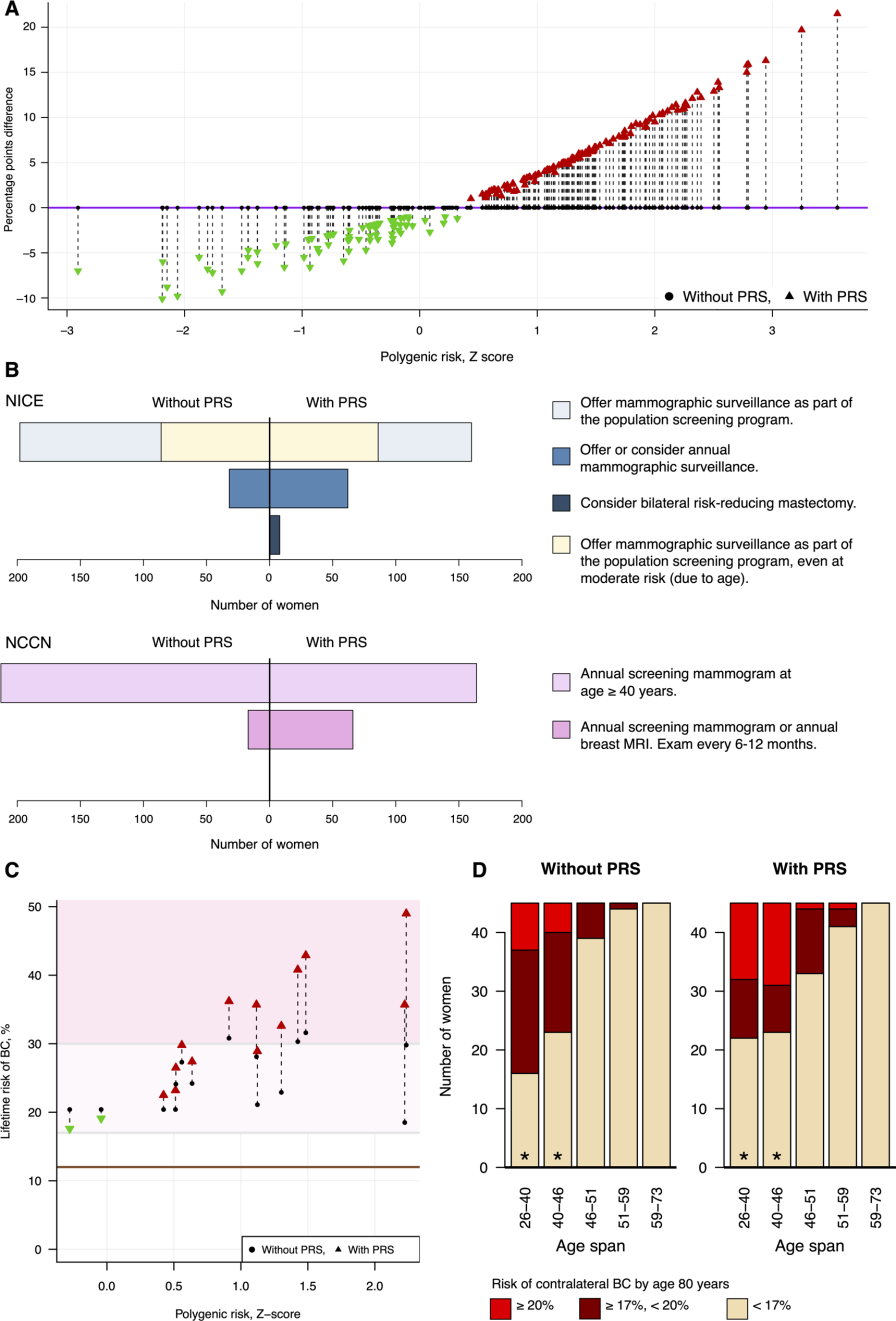
**Panel A**: lifetime risk estimations in women with no pathogenic variant calculated without (points) and with (triangles) PRS_313_. The Y axis displays the difference in absolute risk. Vertical dashed lines connect results without and with PRS_313_, making every arrow correspond to one patient. Only differences of 2% points or more are depicted with triangles. **Panel B**: shows the recommended follow-up care according to NICE and NCCN guidelines, based on risk estimations made without and with PRS_313_ (*n* = 230). Of the 230, 86 women were younger than 40 or older than 59 years and would be offered surveillance as part of the population screening program, even if assigned a moderate risk (NICE, top bars, beige color). **Panel C**: displays individual lifetime risk estimates for women carrying pathogenic variants in moderate-penetrance genes (*ATM*, *CHEK2*, *RAD51C*, *RAD51D*), calculated without (points) and with (triangles) the inclusion of PRS_313_. The shaded background shows the thresholds in the NICE guidelines; 17% and 30%. **Panel D**: presents contralateral BC risk estimates by age 80 for women without a pathogenic variant, calculated without and with the inclusion of PRS_313_. Women are stratified by age group, with color coding to indicate risk categories based on thresholds 17% and 20%

To examine whether incorporation of PRS would impact follow-up care, we reassessed the women using family history and tumor pathology, *without* and *with* inclusion of PRS_313_. The estimated lifetime risk changed risk by 5% points or more in 38% of women (Fig. 2A). The largest shift was from 18 to 40%. Recommended follow-up changed for 23% (*n* = 53) and 32% (*n* = 74) of women based on NCCN and NICE guidelines, respectively, with 22% (NCCN) and 28% (NICE) moving to higher risk categories, and 1% (NCCN) and 5% (NICE) moving to lower categories (Suppl. Figure S3). According to the patients’ age and NICE guidelines, the reevaluation suggested additional annual mammograms for 55 women and consideration of risk-reducing mastectomy for 8 women (Fig. 2B).

For women with a pathogenic variant in a moderate-penetrance gene (*n* = 15), 27% and 20% were reclassified. With risk estimates ranging from 33 to 49%, bilateral mastectomy should be raised as a risk-reducing strategy with these women, according to NICE (Fig. 2C). The average difference in absolute risk with PRS_313_ was 7% (95% CI [3, 10]), with the largest individual risk increase from 30 to 49%. All women with a pathogenic variant in a high-penetrance gene remained in the highest risk class, ranging from 59 to 90% lifetime risk.

### Contralateral risk of BC

We estimated contralateral BC risk by age 80 for women without pathogenic variants (*n* = 229 younger than 80). PRS_313_ changed the risk estimates for 15% of women by at least 5% points; 7% increased, and 8% decreased. The largest increase was from 18 to 26%. Since the risk of contralateral BC foremost depends on age, we stratified our analysis (Fig. 2D). In the younger age groups, 26–46 years of age, the risk estimates made without and with PRS_313_ were significantly different, with a larger proportion in the highest risk class (*P* = 2e-3). In the older age groups, the baseline risk was already low.

Contralateral breast cancer risk estimates for women with a pathogenic variant seemed largely driven by age and the pathogenic variant (Suppl. Figure S4), but the numbers were low (*n* = 32). PRS_313_ changed the estimate by 5% points or more in 15% of cases.

## Discussion

A PRS has potential to improve BC screening programs for the general population. Our observation of the highest PRS_313_ in women with contralateral BC reflects the ability of PRS_313_ to identify women with the highest risk.

In our extended genetic analysis of patients undergoing evaluation for familial BC, women with moderate- or high-penetrant pathogenic variant also showed an increased PRS_313_, on average in the 70:th to 80:th percentile. This underscores the importance of considering the PRS_313_ in evaluation of families with pathogenic variants in moderate-penetrance genes. This also indirectly highlights the need to consider PRS_313_ when pathogenic variants in moderate-penetrance genes are discovered incidentally or through screening, especially in the absence of family history of BC. To estimate the risk of BC, PRS_313_ and pathogenic variants should be evaluated jointly. These results are real-world examples, perfectly in line with results from population-based studies, showing the effect of common variants on penetrance of seemingly monogenic diseases [6].

The predictive capacity of PRS-based risk estimates has been validated in prospective cohort studies [13, 14]. The precision, however, varies with ancestry and has proved less accurate in people of ancestry outside white European populations, likely due to their underrepresentation in GWAS [18–21]. As of 2024, the NCCN discourage the routine use of PRS in BC risk assessment, stressing the need of further validation [19]. Ongoing research will help elucidate the clinical role of PRS in risk reduction efforts [20–22].

In patients ascertained to our center for familial BC, the PRS_313_ is significantly higher than in the general population. Omitting the PRS in this population will underestimate the risk even when the family history is accounted for. This is reflected in that a significant proportion of women would have their screening recommendations changed if PRS_313_ was incorporated in their evaluation. As previously shown by others, the family history and PRS are complementary [5, 13, 15, 17, 23].

This study benefits from detailed pathology data and family histories obtained during clinical genetic counseling. However, it is limited to women already diagnosed with breast cancer, and risk predictions are constrained by missing information on relevant factors such as hormone replacement therapy. Additional limitations include the lack of HER2 status data (except for women with triple-negative tumors) and tumor staging. The predictive performance of PRS_313_ is known to vary across different ancestral backgrounds, underscoring the importance of accounting for ancestry in clinical risk prediction applications [24, 25]. Our findings are therefore most relevant to populations in Sweden with predominantly European ancestry. However, the generalizability of our results beyond this group is limited by the inherent ancestry-related constraints of the PRS_313_. While the control group has previously been described as representative of the general Swedish population, individual cancer histories are not available, and average cancer incidence is assumed rather than observed. Despite these limitations, the study offers valuable real-world evidence from a clinically relevant cohort, integrating both rare and common genetic risk factors.

In conclusion, incorporating PRS_313_ into clinical sequencing enables efficient collection of necessary data without requiring both SNP arrays for PRS calculation and sequencing for pathogenic variants. To fully capture the heritable risk of BC, it is essential to jointly consider both pathogenic variants and common risk alleles.

## Materials and methods

### Study participants

Families with familial BC were recruited retrospectively at the Department of Clinical Genetics, Uppsala University Hospital, Uppsala, Sweden, in accordance with the National Clinical Cancer Care Guidelines [3]. Inclusion criteria are provided in the supplementary methods. In brief, all women had a diagnosis of BC, with early disease onset or affected family members. BC in this study refers primarily to invasive BC but may include ductal carcinoma in situ. Contralateral BC was considered as two separate events. Women with triple-negative BC were included regardless of age.

### Population controls

The SweGen dataset represents a cross-section of 1,000 individuals (506 men and 494 women) from the Swedish population, all of European ancestry [18]. Individual-level disease information is unavailable. The median sampling age of 65 years may limit its representation of the genetic background of recent migrants among our patients.

### Targeted sequencing

DNA extracted from blood samples from all patients was analyzed using a hybrid capture assay designed to capture clinically relevant cancer susceptibility genes and PRS variants, including breast cancer susceptibility genes *ATM*, *BARD1*, *BRCA1*, *BRCA2*, *CDH1*, *CHEK2*, *PALB2*, *PTEN*, *RAD51C*, *RAD51D*, *STK11*, and *TP53* (Suppl. Tables S2, S3-S4). Other genes were not considered in this study. Details of the hybrid capture assay design, library preparation, sequencing, variant identification and classification, genotyping of PRS variants, and quality control procedures are provided in the supplementary methods and supplementary tables S5-S8, S9. The analyses were performed at BRCA-lab, Department of Clinical Sciences, Lund University, as part of clinical screening of cancer susceptibility genes in women referred due to personal or family history of breast cancer, as described above. For each patient, only the genes specified in the clinical referral were analyzed for pathogenic variants. Patients with no pathogenic variant in the genes specified in the referral were defined as panel negative.

For simplicity in this manuscript, likely pathogenic variants were referred to as ‘pathogenic variants’. Established BC genes were classified as high-penetrance (*BRCA1*, *BRCA2*, and *PALB2*) or moderate-penetrance (*ATM*, *CHEK2*, *RAD51C* and *RAD51D*). Low-penetrance risk alleles in these genes (e.g. *CHEK2* p.Ile157Thr, p.Thr476Met, and p.Ser428Phe) were not reported clinically and were not included as pathogenic variants in this study (p.Ile157Thr is, however, included in PRS_313_). No patient with double pathogenic variants in high- or moderate penetrance genes were included.

### Score calculation

The PRS_313_ for overall BC was calculated for all individuals using previously established weights and methods (Suppl. Figure S5) [8, 26] and normalized to a Z score using CanRisk parameters for BCAC 313 (µ = -0.424, sigma = 0.611) [17]. Missing genotype calls were imputed using expected scores based on the risk alleles’ weight and population allele frequency.

### Risk calculations

Contralateral and lifetime risks were assessed using BOADICEA version 6 [17], including family history and tumor pathology, both without and with PRS_313_. To estimate hypothetical lifetime risks, the proband’s age was set to 20 years, and their BC diagnosis was removed, as previously described [26]. For contralateral risk predictions, the proband’s age was adjusted to the age at first BC diagnosis, with any contralateral BC ages omitted.

## Supplementary Information

Below is the link to the electronic supplementary material.

**Supplementary Material 1**: **Supplementary table S1**: The table presents the characteristics of included patients, including genetic screening results, age at first breast cancer diagnosis, presence of contralateral disease, and occurrence of triple-negative cases. **Supplementary table S2**: Table S2 presents the number of subjects sequenced and those with positive findings for each established breast cancer gene. **Supplementary table S9**: The table provides an overview of the impact of missing data on final scores.

Supplementary Material 2

## Data Availability

No datasets were generated or analysed during the current study.

## Abbreviations

BC: Breast cancer
CI: Confidence interval
PRS: Polygenic risk score
SD: Standard deviation
Suppl: Supplementary
