# Supplementary material for: Polygenic scores in Familial breast cancer cases with and without pathogenic variants and the risk of contralateral breast cancer
Source: Breast Cancer Res. 2025 Sep 8;27:160. doi: 10.1186/s13058-025-02107-5 (PMC12418628; doi:10.1186/s13058-025-02107-5)
Supplement: Supplementary file 1 — Supplementary Material 1: Supplementary table S1: The table presents the characteristics of included patients, including genetic screening results, age at first breast cancer diagnosis, presence of contralateral disease, and occurrence of triple-negative cases. Supplementary table S2: Table S2 presents the number of subjects sequenced and those with positive findings for each established breast cancer gene. Supplementary table S9: The table provides an overview of the impact of missing data on final scores. [file 13058_2025_2107_MOESM1_ESM.docx]

Supplementary information for:

**Polygenic scores in familial breast cancer cases with and without pathogenic variants and the risk of contralateral breast cancer**

# SUPPLEMENTARY RESULTS

| **A** | **B** |
| --- | --- |
| **** | **** |

**Supplementary figure S1**

Principal component analysis (PCA) illustrating the genetic ancestry of the study participants (n = 262), shown as black plus signs (+), projected onto reference individuals from the 1000 Genomes Project Phase 1 (n = 2,504, colored points). Each point represents one individual. Colors indicate broad continental ancestry. Panels A and B show principal components 1 vs. 2, and 3 vs. 4, respectively. The PCA was based on 236 overlapping biallelic SNPs shared between the study dataset (based on the PRS313 panel) and the 1000 Genomes reference panel. Ancestry inference was based solely on genetic data but the limited resolution is due to the low number of SNPs in the analysis. Abbreviations: AFR Africa, AMR America, EAS East Asia, EUR Europe, SAS South Asia.

**Supplementary table S1**

|  |  |  |
| --- | --- | --- |
| **Patient characteristics** | | |
| **Included subjects** | n | 262 |
|  |  |  |
| **Panel screening negative** | Proportion | 88% |
|  | n | 230 |
|  |  |  |
|  |  |  |
| **Panel screening positive** | Proportion | 12% |
|  | n | 32 |
|  |  |  |
|  |  |  |
| **Age at first BC** | Mean (Median) | 50 (48) |
|  | Range | 24-80 |
|  |  |  |
|  |  |  |
| **Contralateral BC** | Proportion | 14% |
|  | n | 36 |
|  |  |  |
|  |  |  |
| **Age at contralateral BC** | Mean (Median) | 61 (62) |
|  | Range | 28-77 |
|  |  |  |
|  |  |  |
| **Triple-negative BC** | Proportion | 13% |
|  | n | 44 |
|  |  |  |

Abbreviations: BC, breast cancer.

The table presents the characteristics of included patients, including genetic screening results, age at first breast cancer diagnosis, presence of contralateral disease, and occurrence of triple-negative cases.

**Supplementary figure S2**

Histogram showing the age of first breast cancer (n=262).

**Supplementary table S2**

|  |  | |
| --- | --- | --- |
| **Sequenced breast cancer genes** | | |
| **Gene** | **Number of sequenced subjects** | **Number of positive subjects (‰)** |
| *ATM* | 262 | 1 (4) |
| *BARD1* | 11 | 0 (0) |
| *BRCA1* | 262 | 11 (42) |
| *BRCA2* | 262 | 5 (19) |
| *CDH1* | 10 | 0 (0) |
| *CHEK2* | 262 | 12 (46) |
| *PALB2* | 262 | 1 (4) |
| *PTEN* | 6 | 0 (0) |
| *RAD51C* | 51 | 1 (4) |
| *RAD51D* | 51 | 1 (4) |
| *STK11* | 4 | 0 (0) |
| *TP53* | 253 | 0 (0) |

Table S2 presents the number of subjects sequenced and those with positive findings for each established breast cancer gene.

**Supplementary figure S3**

Risk classification of women without pathogenic variants (n=230), done without and with PRS_313_, based on guidelines from the National Comprehensive Cancer Network (NCCN) and the National Institute for Health and Care Excellence (NICE). Each box represents the fraction of patients in each risk category, which correspond to recommendations for preventive surveillance.

| **A** | **B** |
| --- | --- |
|  |  |

**Supplementary figure S4**

(A) Estimated absolute risk of contralateral breast cancer by age 80 in women with a pathogenic variant, calculated without (points) and with (triangles) the inclusion of PRS_313_. Patients with a pathogenic variant in a high-penetrance gene (*BRCA1*, *BRCA2*, *PALB2*) are highlighted with blue outlines. The x-axis represents the age at first breast cancer diagnosis; The y-axis displays the absolute risk of contralateral breast cancer by age 80. Only patients with a risk difference of ≥2 percentage points are marked with triangles. (B) The x-axis displays the polygenic risk score (Z score), and the y-axis shows the difference in absolute risk estimates with and without PRS_313_. (A and B) Vertical dashed lines connect each patient’s risk estimate without and with PRS313; each arrow represents one patient.

# SUPPLEMENTARY METHODS

**Study participants**

Families were recruited at the Department of Clinical Genetics, Uppsala University hospital, Uppsala, Sweden. Inclusion criteria for genetic evaluation for familial BC were: (i) BC at age 40 or younger; (ii) BC at or before age 50 with a first- or second-degree relative diagnosed with BC*; or (iii) BC at or before age 60 with two first- or second-degree relatives diagnosed with BC*. All women had a diagnosis of BC. Ovarian cancer or prostate cancer before age 65 also qualified as BC* in these criteria. BC in this study refers primarily to invasive BC but may include ductal carcinoma in situ. Contralateral BC was considered as two separate events. Women with triple-negative BC were included regardless of age. The study was approved by the Swedish Ethical Review Authority (Dnr. 2021-05879-01).

**Population controls**

The SweGen dataset provides a representative cross-section of the Swedish population (Ameur et al, 2017). It was originally drawn from the Swedish Twin Registry and the Northern Sweden Population Health Study, selecting 1,000 individuals born in Sweden through principal component analysis to capture the key genetic structure of the country. Due to the age of these cohorts, SweGen may not fully reflect the genetic background of more recent migrants in our patient group. Family history or disease status was not considered when selecting the 1,000 participants, whose median sampling age was 65 years. The dataset was generated by Science for Life Laboratory, and its whole-genome sequencing data is available to the research community.

**Hybrid capture library preparation and sequencing**

DNA was extracted from peripheral blood lymphocytes using Chemagic Prime 4 (DNA Blood 2k Kit H24, ©Revvity Inc). Libraries of genomic fragments were prepared from DNA using KAPA HyperPlus (Kit KK8514, Roche) according to manufacturer’s instructions with these modifications: 1) Fragmentation conditions were adjusted to 30°C for 7 minutes 30 seconds. 2) Custom short adapters that contain the binding sites for Illumina sequencing primers were used in the ligation reaction (table S5). 3) The adapter-ligated library was amplified for five PCR cycles using custom primers containing 8 nt library-specific index sequences and the Illumina P7 or P5 sequences required for the library to bind and generate clusters on the sequencing flow cell (table S6).

Hybrid capture was performed using a Twist Custom Panel (Twist Bioscience) according to manufacturer’s instructions with the following modifications: 1) Sixteen sample libraries were pooled before hybrid capture, 282ng DNA from each library.

The sixteen-sample hybrid-capture pools were combined for sequencing and spiked with 1% PhiX as quality control (Illumina, FC-110-3001). Up to 64 barcoded sample libraries were sequenced together, depending on sequencing instrument and reagent kit, aiming for about 300x to 400x mean sequence coverage over the target genomic regions. An aliquot was diluted to optimal concentration, loaded on a flowcell and paired-end sequenced with 2x150bp reads on an Illumina NextSeq 1000 (P2 or P1 reagent kits, Illumina 20046813, 20050264) or NextSeq 500 (high or mid output v2.5 reagent kits, Illumina 20024908, 20024905).

**Hybrid capture design**

The Twist custom hybridization assay was designed to target 50 genes. For 42 genes the design target includes exons, introns and 5kb up- and downstream of RefSeq coding transcripts for the gene. For introns longer than 11kb, the central part was excluded from the design target, keeping 5kb at each end of the intron. For five genes the design target includes only coding exons plus 20bp flanking introns. For the TERT gene, only the proximal promoter region is included, for GREM1 only the 5’ regulatory region affected by duplications identified in some patients with hereditary colorectal cancer syndromes (Jaeger et al., 2012, Rohlin et al 2015) and for CDK4 only exon 2 is included in the design target. Some target regions outside coding exons, mainly repetitive elements and regions of low complexity, were excluded to avoid excessive off-target capture. For details of the assay design per gene, see table S3. The total length of intended target genomic regions including repetitive elements and regions of low complexity was 2 572 391 bp of which 75.1% was directly covered by probes in the final assay design. The assay also includes probes designed to target the 313 genetic variants included in the PRS of Mavaddat et al. 2019 (table S4) and 63 variants for verification of sample identity and sex (see section ‘smMIPs assay for sample identity verification’ below).

**Hybrid capture data analysis**

Illumina instrument basecalls were separated based on library-specific adapter index sequences (demultiplexed) and converted to fastq format using Picard tools ExtractIlluminaBarcodes and IlluminaBasecallsToFastq (v2.24.2 or v3.0.0, http://broadinstitute.github.io/picard). Default settings were used except for MAX_MISMATCHES 2, MIN_MISMATCH_DELTA 2, MAX_NO_CALLS 2 and MIN_QUAL 0.

Sequences were aligned to the human reference genome and genetic variants detected as described in Öfverholm et al (2023) with minor changes detailed below. Briefly, Novoalign (Novocraft Technologies Sdn Bhd, Petaling Jaya, Selangor, Malaysia) was used for alignment, Genome Analysis Toolkit (GATK, McKenna et al. 2010, DePristo et al., 2011) to identify single nucleotide substitutions and small insertions and deletions, and a combination of Manta (Chen et al. 2016), XHMM (Fromer et al. 2012) and Melt (Gardner et al. 2017) for identification of larger structural variants. Variants were reviewed in Integrative Genomics Viewer (IGV, Robinson et al 2011) and variants classified as likely pathogenic or pathogenic were confirmed using smMIPs, Sanger sequencing or MLPA (see below). Software versions and settings are the same as in Öfverholm et al (2023) except for these differences: 1) Trimmomatic version 0.39 (Bolger et al 2014) was used to trim adapters and low-quality bases before alignment. For adapter trimming, Trimmomatic settings were MINLEN:30 and 3:12:7:1:true. For quality trimming, Trimmomatic settings were MINLEN:30 and MAXINFO:30:0.25. 2) Novoalign version 4.00 was used for alignment. 3) Picard version 2.20.8 was used for MarkDuplicates. 4) Local realignment was performed using GATK version 3.8.1.0. 5) GATK version 4.1.8.1 was used for DepthOfCoverage. 6) Small variant calling was done using GATK version 3.8. Genotype calls for the 313 variants included in the PRS were made using HaplotypeCaller of GATK version 4.5.0.0.

Pathogenicity classification of variants was performed as described in Öfverholm et al (2023).

**Variant confirmation using smMIPs and Sanger sequencing**

The first time a pathogenic or likely pathogenic variant was identified in the lab using the hybrid capture assay, it was confirmed using an orthogonal method, either single-molecule Molecular Inversion Probes (smMIPs, Hiatt et al 2013), Sanger sequencing or Multiplex Ligation-dependent Probe Amplification (MLPA, Schouten et al 2002).

**smMIPs library preparation and sequencing**

Library Preparation for the smMIPs assays was performed as described for “manual experiments” in Eijkelenboom (2016) with these modifications: 1) Amplification was done using custom primers with 8 nucleotide library-specific index sequences (table S7). 2) PCR products were purified twice with 0.9x volume HighPrep beads (MagBio).

**smMIPs design**

The smMIPs consist of a central 30 nt backbone and target specific extension and ligation arms (16 nt to 29 nt) with random single molecule tags in between (4 nt). We used the MIPgen pipeline version 1.1 (Boyle et al 2014) to design smMIPs targeting coding exons plus 20bp flanking introns of 19 genes. The length of targeting arms plus insert region to be captured was restricted to 150-170 nt ( min_capture_size 150, max_capture_size 170) and we inserted random single molecule tags of 4 nt on each side of the central backbone ( tag_sizes 4,4). The smMIPs were designed to fully cover both strands to ensure that all target regions were captured by two smMIPs ( double_tile_strands_separately). We used the SVR scoring method ( score_method svr) and Tandem Repeats Finder was enabled to flag MIPs with tandem repeats in the MIP arms. Known single nucleotide variant (SNP) alleles in the ExAC database (version 0.3.1, Karczewski et al 2017) with overall allele frequency above 0.05 and SNP alleles in the SweGen database (version 2016122, Ameur et al 2017) with allele frequency above 0.02 were taken into account in the design process ( snp_file). In a few cases SNPs in targeting arms could not be avoided and then we designed separate smMIPs to capture each allele. For BRCA1 and BRCA2 and the CHEK2 variant c.1100del we used the smMIPs designed by Neveling et al (2017) modified to include dual 4 nt random single molecule tags. We also designed smMIPs to target 63 variants for verification of sample identity and sex (see section ‘smMIPs assay for sample identity verification’ below). Probes were pooled at equimolar concentrations in different combinations to construct assays targeting subsets of genes. In total, our smMIPs assay pools are based on a set of 2429 smMIPs (table S8).

**smMIPs data analysis**

Basecalling and demultiplexing was performed as for hybrid capture data. JSI SEQUENCE Pilot version 4.4.0 Build 508 module SEQNEXT (JSI medical systems GmbH, Ettenheim Germany) was used for sequence alignment and variant calling of smMIPs data.

**Sanger sequencing**

Sanger sequencing was performed with Big Dye Terminator v1.1 Cycle Sequencing Kit (Applied Biosystems, Foster City, CA, USA) and ran on a SeqStudio Genetic Analyzer, Applied Biosystem. Sequencher® version 5.0 DNA sequence analysis software (Gene Codes Corporation, Ann Arbor, MI USA) was used to evaluate chromatograms.

**MLPA**

Some copy-number altering structural variants were confirmed using SALSA MLPA Probemix kits (Kits P002 for BRCA1 variants, P045 for BRCA2, P041 or P042 for ATM, P056 for TP53, P083 for CDH1, P101 for STK11, P190 for CHEK2, P225 for PTEN, P260 for PALB2, RAD51C and RAD51D and P489 for BARD1). The assays were performed according to the manufacturer´s instructions (MRC-Holland, Amsterdam, The Netherlands) and analysed using GeneMarker v1.6 (Softgenetics, PA, USA).

**smMIPs assay for sample identity verification**

An aliquot of each patient’s blood sample was separated when the sample was received at the laboratory. DNA was extracted and a separate library was prepared and sequenced using an smMIPs assay targeting 63 variants. The variants include 51 variants chosen to cover all autosomes and have high minor allele frequency in the Swedish population (Stranneheim et al, 2021), a set of 9 variants from an earlier smaller in-house identity verification panel and 3 variant sites for sex determination (Kim et al 2010). The same set of variants were also included as targets in the hybrid capture assay and smMIPs assays for variant confirmation. The genotype profile called from the hybrid capture, smMIPs assays for variant confirmation and smMIPs assay for sample identity verification were compared to detect any potential sample mix-ups in the laboratory.

**PRS panel performance**

We quality controlled the sequencing data for the 313 PRS targets. First, we assessed the level of missing data, which was found to be negligible, with no meaningful impact on the final PRS calculations. The maximum number of missing genotype calls per sample was 1 out of 313. Any potential ambiguities in transitioning from array-based to sequencing-based variant calls were resolved using previously described methods (Baliakas et al, 2024). The strong correlation between observed and expected risk allele frequencies supports the proper matching of risk alleles (Suppl. Figure S5). To calculate the PRS, we summed the corresponding weights of the risk alleles at the 313 genomic markers, as specified by Mavaddat et al, 2019. Scores for missing calls were imputed using the population allele frequencies.

| **A** | **B** |
| --- | --- |
|  |  |
| **Supplementary figure S5**  Panels A and B show the expected effect allele frequencies from the CanRisk score file compared to observed allele frequencies in (A) population controls (SweGen, n=1000) and (B) patients evaluated for familial breast cancer (n=262). | |

**Sensitivity analysis**

We analyzed the impact of missing variant calls (Supplementary Table S9). With a maximum of one missing genotype per individual, the difference between raw and imputed PRS was 0.03 (Z score), corresponding to less than a 1% change in absolute lifetime breast cancer risk. We proceeded with the PRS calculated using imputed genotypes.

**Supplementary table S9**

|  |  |  |  |
| --- | --- | --- | --- |
|  |  | **Population controls, SweGen** | **Familial breast cancer** |
| **Number of subjects** |  | 1000 | 262 |
|  |  |  |  |
| **Number of missing genotype calls per sample** | Mean (SD) | 0.8 (0.73) | 0.06 (0.24) |
|  | Range | 0-4 | 0-1 |
|  |  |  |  |
|  |  |  |  |
| **Raw PRS, calculated with missing genotypes** | Mean (SD) | -0.41 (0.60) | -0.03 (0.67) |
|  | Range | -2.7 – 1.4 | -2.2 – 1.7 |
|  |  |  |  |
|  |  |  |  |
| **Imputed PRS, calculated with missing genotypes substituted for expected dosage** | Mean (SD) | -0.42 (0.59) | -0.04 (0.67) |
|  | Range | -2.7 – 1.3 | -2.2 – 1.7 |
|  |  |  |  |
|  |  |  |  |
| **Difference between raw and imputed PRS** | Mean (SD) | 0.01 (0.01) | 0.00 (0.01) |
|  | Range | -0.08 – 0.07 | -0.03 – 0.03 |
|  |  |  |  |

The table provides an overview of the impact of missing data on final scores.

**PRS in the SweGen database**

For comparisons, we calculated PRS_313_ for 1000 Swedish population controls, as described before^2^. In summary, the observed allele frequencies were close to expected, and imputing missing data had no significant effect on PRS. The Swedish control population was comparable to European ancestry populations, supporting its use in comparative analyses.

**Risk calculations**

Pedigree information was collected by trained clinical geneticists and specialized nurses, and typically retrieved unchanged from medical records. Contralateral and lifetime breast cancer risks were calculated with and without PRS_313_ using BOADICEA version 6 and the CanRisk Batch Tool, as previously described ^2^. Variables for age of first live birth and parity were not used.

Contralateral risk estimates

For contralateral risk, the proband’s age was set to the age at first breast cancer diagnosis, and contralateral breast cancer information was removed. Family members born after the diagnosis were excluded, and ages adjusted. Test results from panel screening were included.

Lifetime risk estimates

For hypothetical lifetime risk estimates, the proband’s age was set to 20 years, with the year of birth adjusted accordingly. Breast cancer diagnoses and pathology information were erased, and panel screening results were not included.

**Ancestry**

Ancestry information was estimated using principal component analysis in the presence of 2,504 samples from the international 1,000 genomes project. The analysis was restricted to biallelic SNPs overlapping the PRS313 and the 1,000 genomes project dataset.

**Statistical Analysis**

The Wilcoxon Rank Sum test was employed to compare average PRS between two groups. This non-parametric test was chosen due to the relatively small sample size, as it reduces the risk of false positives when the assumption of normality is violated and is less affected by potential outliers. Fisher's exact test was used for analyzing simple proportions. All methods were carried out using the stats package in R version 4.3.2.

**REFERENCES**

Ameur A, Dahlberg J, Olason P, et al. SweGen: a whole-genome data resource of genetic variability in a cross-section of the Swedish population. Eur J Hum Genet. 2017;25(11):1253-1260. doi:10.1038/ejhg.2017.130. PMID: 28832569

Baliakas, P. et al. Integrating a Polygenic Risk Score into a clinical setting would impact risk predictions in familial breast cancer. J Med Genet 61, 150–154 (2024). PMID: 37580114

Bolger AM, Lohse M, Usadel B. Trimmomatic: a flexible trimmer for Illumina sequence data. Bioinformatics. 2014;30(15):2114-2120. doi:10.1093/bioinformatics/btu170. PMID: 24695404

Boyle EA, O'Roak BJ, Martin BK, Kumar A, Shendure J. MIPgen: optimized modeling and design of molecular inversion probes for targeted resequencing. Bioinformatics. 2014;30(18):2670-2672. doi:10.1093/bioinformatics/btu353. PMID: 24867941

Chen X, Schulz-Trieglaff O, Shaw R, et al. Manta: rapid detection of structural variants and indels for germline and cancer sequencing applications. Bioinformatics. 2016;32(8):1220-1222. doi:10.1093/bioinformatics/btv710. PMID: 26647377

DePristo MA, Banks E, Poplin R, et al. A framework for variation discovery and genotyping using next-generation DNA sequencing data. Nat Genet. 2011;43(5):491-498. doi:10.1038/ng.806. PMID: 21478889

Eijkelenboom A, Kamping EJ, Kastner-van Raaij AW, et al. Reliable Next-Generation Sequencing of Formalin-Fixed, Paraffin-Embedded Tissue Using Single Molecule Tags. J Mol Diagn. 2016;18(6):851-863. doi:10.1016/j.jmoldx.2016.06.010. PMID: 27637301

Fromer M, Moran JL, Chambert K, et al. Discovery and statistical genotyping of copy-number variation from whole-exome sequencing depth. Am J Hum Genet. 2012;91(4):597-607. doi:10.1016/j.ajhg.2012.08.005. PMID: 23040492

Gardner EJ, Lam VK, Harris DN, et al. The Mobile Element Locator Tool (MELT): population-scale mobile element discovery and biology. Genome Res. 2017;27(11):1916-1929. doi:10.1101/gr.218032.116. PMID: 28855259

Hiatt JB, Pritchard CC, Salipante SJ, O'Roak BJ, Shendure J. Single molecule molecular inversion probes for targeted, high-accuracy detection of low-frequency variation. Genome Res. 2013;23(5):843-854. doi:10.1101/gr.147686.112. PMID: 23382536

Jaeger E, Leedham S, Lewis A, et al. Hereditary mixed polyposis syndrome is caused by a 40-kb upstream duplication that leads to increased and ectopic expression of the BMP antagonist GREM1. Nat Genet. 2012;44(6):699-703. Published 2012 May 6. doi:10.1038/ng.2263. PMID: 22561515

Karczewski KJ, Weisburd B, Thomas B, et al. The ExAC browser: displaying reference data information from over 60 000 exomes. Nucleic Acids Res. 2017;45(D1):D840-D845. doi:10.1093/nar/gkw971. PMID: 27899611

Kim JJ, Han BG, Lee HI, Yoo HW, Lee JK. Development of SNP-based human identification system. Int J Legal Med. 2010;124(2):125-131. doi:10.1007/s00414-009-0389-9. PMID: 19921517

Mavaddat N, Michailidou K, Dennis J, et al. Polygenic Risk Scores for Prediction of Breast Cancer and Breast Cancer Subtypes. Am J Hum Genet. 2019;104(1):21-34. doi:10.1016/j.ajhg.2018.11.002. PMID: 30554720

McKenna A, Hanna M, Banks E, et al. The Genome Analysis Toolkit: a MapReduce framework for analyzing next-generation DNA sequencing data. Genome Res. 2010;20(9):1297-1303. doi:10.1101/gr.107524.110. PMID: 20644199

Neveling K, Mensenkamp AR, Derks R, et al. BRCA Testing by Single-Molecule Molecular Inversion Probes. Clin Chem. 2017;63(2):503-512. doi:10.1373/clinchem.2016.263897. PMID: 27974384

Öfverholm A, Törngren T, Rosén A, et al. Extended genetic analysis and tumor characteristics in over 4600 women with suspected hereditary breast and ovarian cancer. BMC Cancer. 2023;23(1):738. Published 2023 Aug 10. doi:10.1186/s12885-023-11229-y. PMID: 37563628

Robinson JT, Thorvaldsdóttir H, Winckler W, et al. Integrative genomics viewer. Nat Biotechnol. 2011;29(1):24-26. doi:10.1038/nbt.1754. PMID: 21221095

Rohlin A, Eiengård F, Lundstam U, et al. GREM1 and POLE variants in hereditary colorectal cancer syndromes. Genes Chromosomes Cancer. 2016;55(1):95-106. doi:10.1002/gcc.22314. PMID: 26493165

Schouten JP, McElgunn CJ, Waaijer R, Zwijnenburg D, Diepvens F, Pals G. Relative quantification of 40 nucleic acid sequences by multiplex ligation-dependent probe amplification. Nucleic Acids Res. 2002;30(12):e57. doi:10.1093/nar/gnf056. PMID: 12060695

Stranneheim H, Lagerstedt-Robinson K, Magnusson M, et al. Integration of whole genome sequencing into a healthcare setting: high diagnostic rates across multiple clinical entities in 3219 rare disease patients. Genome Med. 2021;13(1):40. Published 2021 Mar 17. doi:10.1186/s13073-021-00855-5. PMID: 33726816
